# New tools for old problems — comparing drone- and field-based assessments of a problematic plant species

**DOI:** 10.1007/s10661-021-08852-2

**Published:** 2021-01-27

**Authors:** Jens Oldeland, Rasmus Revermann, Jona Luther-Mosebach, Tillmann Buttschardt, Jan R. K. Lehmann

**Affiliations:** 1 Insitute of Plant Sciences and Microbiology, University of Hamburg, Ohnhorststr. 18, 22609 Hamburg, Germany; 2 Netzwerk für Angewandte Ökologie, Hamburg, Germany; 3 Institute of Landscape Ecology, University of Münster, Münster, Germany

**Keywords:** Vegetation mapping, Nature conservation, Hallig, OBIA, UAV, Salt marsh, Elymus athericus, Nordstrandischmoor

## Abstract

Plant species that negatively affect their environment by encroachment require constant management and monitoring through field surveys. Drones have been suggested to support field surveyors allowing more accurate mapping with just-in-time aerial imagery. Furthermore, object-based image analysis tools could increase the accuracy of species maps. However, only few studies compare species distribution maps resulting from traditional field surveys and object-based image analysis using drone imagery. We acquired drone imagery for a saltmarsh area (18 ha) on the Hallig Nordstrandischmoor (Germany) with patches of *Elymus athericus*, a tall grass which encroaches higher parts of saltmarshes*.* A field survey was conducted afterwards using the drone orthoimagery as a baseline*.* We used object-based image analysis (OBIA) to segment CIR imagery into polygons which were classified into eight land cover classes. Finally, we compared polygons of the field-based and OBIA-based maps visually and for location, area, and overlap before and after post-processing. OBIA-based classification yielded good results (kappa = 0.937) and agreed in general with the field-based maps (field = 6.29 ha, drone = 6.22 ha with *E*. *athericus* dominance). Post-processing revealed 0.31 ha of misclassified polygons, which were often related to water runnels or shadows, leaving 5.91 ha of *E*. *athericus* cover. Overlap of both polygon maps was only 70% resulting from many small patches identified where *E*. *athericus* was absent. In sum, drones can greatly support field surveys in monitoring of plant species by allowing for accurate species maps and just-in-time captured very-high-resolution imagery.

## Introduction

Drones, or UAS (unmanned aerial systems), are becoming a valuable tool for mapping problematic or endangered plant species (Alvarez-Taboada et al. 2017; Rominger and Meyer 2019). UAS are cost friendly and allow flexible application in space and time, and very-high-resolution (VHR) image data can be acquired within 1 h by a single person, e.g., before or during a field survey. Precise capture of relatively large areas, e.g., up to 400 ha, can be done in one single flight (Samiappan et al. 2017a). Besides regular RGB cameras, multispectral cameras can be mounted, which are now affordable, and allow additional information on vegetation vigor (Oldeland et al. 2017). Constraints like strong winds, rainfall, or snow pose a problem for UAS missions, but this is also true for airplane campaigns. Further limitations are often high geometric errors and high data volumes as the low flying UAS can provide VHR imagery with ground sampling distances of a few centimeters. Still, just-in-time VHR image data can support conservation and natural resource management, e.g., by mapping plant individuals at the scale of a field survey.

The very high spatial resolution and the advantages in terms of flexibility make UAS highly suitable for mapping plant species on a local spatial scale. In particular, invasive plants or plants that tend to encroach within wetlands have been in focus of several recent UAS-based studies. Wan et al. (2014) compared UAS images from 2009 to 2011 to map the encroachment of the C4 grass *Spartina alterniflora* in the Beihai wetland in China. They measured an increase of almost 20% within these 2 years on a scale of 300 ha. *Phragmites australis* is another well-known example for a strongly expanding tall grass species. Others compared different image texture measures (Samiappan et al. 2017a) and multispectral indices (Samiappan et al. 2017b) to map *Phragmites australis* patches in different wetlands of Louisiana, USA. A combination of texture and vegetation indices worked best to capture the extent of *Phragmites* invasion. Hill et al. (2017) demonstrated the utility of UAS for mapping *Iris pseudacorus* at two different lakes in British Columbia, Canada. They compared three different approaches to map the species, i.e., field survey, manual image interpretation, and pixel-based classification. The manual image interpretation yielded the highest accuracies. These studies confirm the applicability of UAS to map plant species in different wetlands using image analysis or manual image interpretation.

*Elymus athericus* (Sea Couch Grass) is a native grass species that occurs in saltmarshes in central Europe. It is a typical species of the high saltmarsh. Its establishment is facilitated by natural sedimentation processes (Nolte et al. 2019). It was observed that *E*. *athericus* is able to partially invade the low marsh overgrowing smaller species (Bakker et al. 2009). When grazing is abandoned or reduced, the species becomes dominant (Nolte et al. 2019, Bakker et al. 2020) and then negatively affects species diversity of plants (Wanner et al. 2014) or halophilic spiders (Pétillon et al. 2005) and reduces the habitat suitability for certain breeding birds (Mandema et al. 2015). Nevertheless, long-term abandoned saltmarshes with high dominance of *E*. *athericus* make an important contribution to diversity if all trophic levels are considered (Rickert et al. 2012, Van Klink et al. 2016) and provide more fish feeding opportunities in winter (Friese et al. 2018). Thus, this type of saltmarsh forms an integral part of saltmarsh conservation (Van Klink et al. 2016). Furthermore, *E*. *athericus* can trap carbon and positively affects C sequestration, thus supporting the climate change mitigation function of saltmarshes (Valéry et al. 2004). Besides these ecological implications, encroachment of *E*. *athericus* can have negative economic impacts as it reduces the yield of meadows and sheep or cattle do not eat the fully grown plants. Consequently, the encroached area is lost for livestock farming. Hence, monitoring of *E*. *athericus* expansion in response to different land uses is an important contribution for decision-making concerning environmental management.

On the Halligen, a type of seasonally inundated islands, in the North Frisian Wadden Sea, Germany, farmers receive funding for appropriate management practices through a conservation covenant scheme called “Hallig Programm.” A scientific monitoring program evaluates the effects of the management on vegetation development in general and in particular on the stands of *E*. *athericus*. On Hallig Nordstrandischmoor, surveys are carried out in intervals of 2 years (Revermann and Luther-Mosebach, 2019). During a field survey, the distribution and cover of *E*. *athericus* is recorded in the field, sketched on aerial photographs, and then later digitized as polygons to support nature conservation offices. The accuracy of the maps depends on aerial imagery which is often not from the same year as the survey but often several years older. Without recent aerial imagery, current *E*. *athericus* patches are difficult to accurately delineate in the field. UAS imagery acquired shortly before or during a field survey could help in precise mapping of these tall grass patches. However, no study evaluated the performance of UAS-based image classification for the case of *E*. *athericus* in saltmarshes in central Europe. In particular, object-based image analysis (OBIA), which, similar to a field surveyor, delivers polygon patches as an output, are highly suitable to analyze VHR imagery (Kucharczyk et al. 2020). However, a comparison of field survey–based maps and OBIA-based maps derived from VHR-UAS-imagery has not been evaluated for the case of *E*. *athericus*. Hence, it is not clear whether conducting UAS missions can improve current approaches for managing *E*. *athericus* in saltmarsh pastures or whether it only adds to the costs and complexity.

The aim of this study is to verify the applicability of UAS images for mapping the distribution of *E*. *athericus* in saltmarshes of the Wadden Sea National Park and adjecent areas, Germany. We apply object-based image analysis and provide a workflow to optimize settings of the image segmentation algorithm and simultaneously compare three different classification algorithms: random forest, elastic net, and artificial neural networks. Finally, we compare the maps resulting from traditional field survey work with OBIA-based maps and discuss the advantages and disadvantages of UAS for supporting vegetation surveys.

## Methods

### Study area

**Fig. 1:**
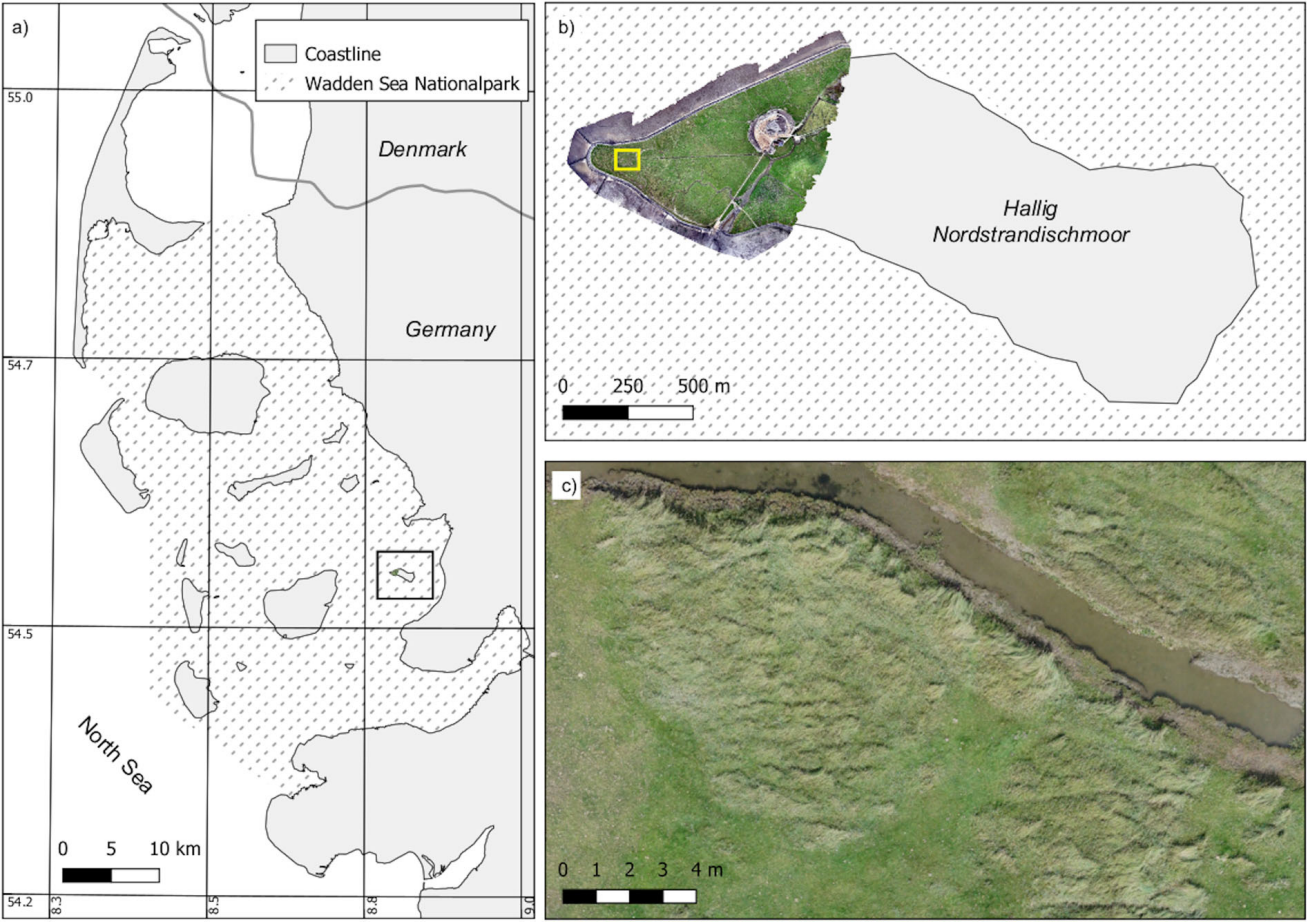
Location of the study area on the Hallig Nordstrandischmoor. The Hallig is located in the Wadden Sea National Park at the North Sea Coast of Germany (**a**). The UAS-orthomosaic was acquired on the western tip of the Hallig (**b**) covering 18 ha. *Elymus athericus* appears in large bright and strongly textured patches (**c**). Wadden Sea boundaries taken from WDPA (UNEP-WCMC and IUCN 2020)

This study took place on the western tip of the Hallig Nordstrandischmoor in the Wadden Sea National Park (Fig. 1a). Hallig is a type of seasonally inundated island at the North Sea coast of Germany. It is typically made of fine-textured marsh sediments from the North Sea and is a remnants of a former coherent marshland. The area is dissected by tidal channels and is surrounded by a low dyke to prevent frequent flooding in summer (Fig. 1b). Vegetation belongs to the phytosociological order *Glauco-Puccinellietalia maritimae* and consists of typical species of the lower and higher saltmarsh like *Elymus athericus*, *Festuca rubra*, *Puccinellia maritima*, and *Limonium vulgare*. Vegetation is patchy with locally dense stands of *E*. *athericus* surrounded by short grazed saltmarsh consisting mainly of *Festuca rubra* and *Puccinellia maritima* (Fig. 1c).

### Field survey

A field survey was conducted in October 2019 after the UAS image acquisition (see below). Vegetation types were mapped according to the TMAP protocol (Petersen et al. 2014), adapted for the federal state of Schleswig-Holstein. In the protocol, the *E*. *athericus* type requires a coverage of that species > 30%. In total, eight different vegetation types were mapped on a scale 1:500 by drawing polygons on printed UAS RGB imagery (Fig. 1b). Next to the mapping of vegetation types the coverage of *E*. *athericus* was estimated in the entire study area and assigned to classes of 10% ranging from 0 to 100%.

For comparison with the results of the UAS-based approach, two measures were calculated: first, for comparison with the vegetation classes generated by the classification of UAS imagery, we used the vegetation types of the survey, e.g., the type “*Elymus athericus* lawn.” Second, the total area covered by *E*. *athericus* was calculated by multiplying the area of the polygon with homogenous vegetation cover with the cover class estimated for that polygon. Thus, we can obtain one figure for the *E*. *athericus* cover of the study area.

### UAS image acquisition

Image data was acquired in August 2019 using a WingtraOne (Wingtra AG, Zurich) platform. This hybrid tail-sitter drone enables image data collection of areas up to 400 ha in a single flight. To generate a broad range of aerial data, both a full-frame Sony RX1RII 42-megapixel RGB and a Micasense RedEdge-MX camera with five narrow bands were used. Accordingly, the survey area (Fig. 1b) was mapped in two single flights: one for RGB and one for multispectral imagery. The forward and sideward image overlap was set to 80% for RGB and 70% for multispectral imagery, respectively. Both flights were carried out at a pre-set altitude of 100 m above ground level, resulting in a ground sample distance (GSD) of approximately 1.4 cm for RGB and 6.8 cm for multispectral imagery (Fig. 1c). For multispectral data post-calibration, a calibrated white reference panel was recorded shortly before and after flight. In addition, an on-board installed downwelling light sensor (DLS) was used to enable improved reflectance calibrations in situations where light conditions are changing in mid-flight, for example, due to temporary clouds. Weather conditions were slightly windy with scattered cloud cover. Both flights were carried out around solar noon to avoid strong cast shadow effects.

### Image data processing

The collected image data allowed us to provide mapping products such as orthomosaics, dense point clouds, and digital surface models. We used the software Pix4Dmapper (Pix4D SA, Lausanne, Switzerland) for the photogrammetric imagery processing and point cloud generation. In case of the multispectral image data, we used a standardized radiometric calibration to correct the image reflectance values according to the calibration target and the DLS. It thus takes into consideration the illumination conditions at the date, time, and location of the image capture as well as the sensor’s characteristics. The calibration panel allows calculating absolute reflectance values which make it possible to compare data coming from different flights. In addition, a Canopy Height Model (CHM) was derived in QGIS (QGIS Development Team 2019) by subtracting the digital (ground) elevation model (DEM) from the elevation of the top of the digital surface model (DSM). Due to the higher optical sensor resolution and thus higher accuracies, we used only the RGB imagery for CHM generation.

### Spectral indices

The UAS also captured multispectral images via the Micasense RedEdge-MX in the wavelengths of the visual, red edge (717 nm center, 10 nm bandwidth) and near-infrared (840 nm center, 40 nm bandwidth). This allowed us to calculate a set of spectral indices. We chose the Normalized Difference Vegetation Index (NDVI) which is a standard vegetation index for characterizing density of green biomass (Tucker 1979). Given the moist character of the ecosystem, its spatial variability, and the various water channels, we further calculated the Normalized Difference Water Index (NDWI) after McFeeters (1996). The NDVI relies on the NIR and red channel, while the NDWI requires green and NIR channels. Both spectral indices were calculated using the command line function “otbcli_RadiometricIndices.bat” of the Orfeo Toolbox Software (McInerney and Kempeneers 2015).

### Structural Feature Set texture

In the drone imagery, *E*. *athericus* patches show strong textural patterns when compared to other grass patches (Fig. 1c). To exploit the textural information, we calculated Structural Feature Set (SFS) texture measures (Huang et al. 2007) based on the RGB imagery. SFS rely on the histograms of the pixels in multiple directions from a central pixel. We computed six parameters based on the histograms: SFS-length, SFS-width, SFS-PSI, SFS-weighted-mean, SFS-ratio, and SFS-SD (standard deviation). The SFS indices are computed from the neighborhood of each pixel. It is possible to change the length of the line (spatial threshold), as well as the maximum difference between a pixel of the line and the pixel at the center of the neighborhood (spectral threshold). Calculations were done using the default values for the command line function “otbcli_SFSTextureExtraction.bat” of the Orfeo Toolbox Software (McInerney and Kempeneers 2015).

### Large-scale mean shift segmentation

To perform object-based image analysis, the raster information has to be separated into polygons of similar spatial and spectral characteristics by image segmentation. The image segmentation was performed on a resampled 12-cm color-infrared (CIR) image, as performance was highly improved when compared to the 1.4-cm resolution and image noise due to the texture of *E*. *athericus* was reduced using coarser images. We used the large-scale mean shift segmentation (LSMS) application provided by the Orfeo Toolbox (McInerney and Kempeneers 2015), command line function “otbcli_LargeScaleMeanShift.bat.” It chains together the four modules for (1) stabilized mean shift smoothing, (2) connected-components segmentation, (3) small region merging, and (4) vectorization. The output is a polygon vector file in which the polygons resemble objects in the image. The LSMS algorithm is explained in more detail in Michel et al. (2015).

The application requires the setting of three parameters, the spatial radius (sp = distance in pixel), the spectral range (sr, distance in Euclidean spectral space), and the minimum size of an object in pixels. As the optimal setting was not known, different combinations of parameter settings were calculated and their respective performance was evaluated. The minimum size parameter was constantly kept at 20 pixels as this was the estimated minimum area of an *Elymus* patch. The spatial radius was calculated for the values 10, 20, and 30, while the spectral range was calculated for 20, 30, and 40. The calculation of the spatial radius = 30 data took extremely long for spectral ranges larger than 20; hence, for sp = 30, only this sr value was calculated. This step yielded nine different segmentation models. After image segmentation, polygons were used to calculate zonal statistics (mean, standard deviation, Gini coefficient) for the prepared image parameters using SAGA-GIS (Conrad et al. 2015). These parameters per polygon were used as model input for the classification.

### Object classification

**Fig. 2:**
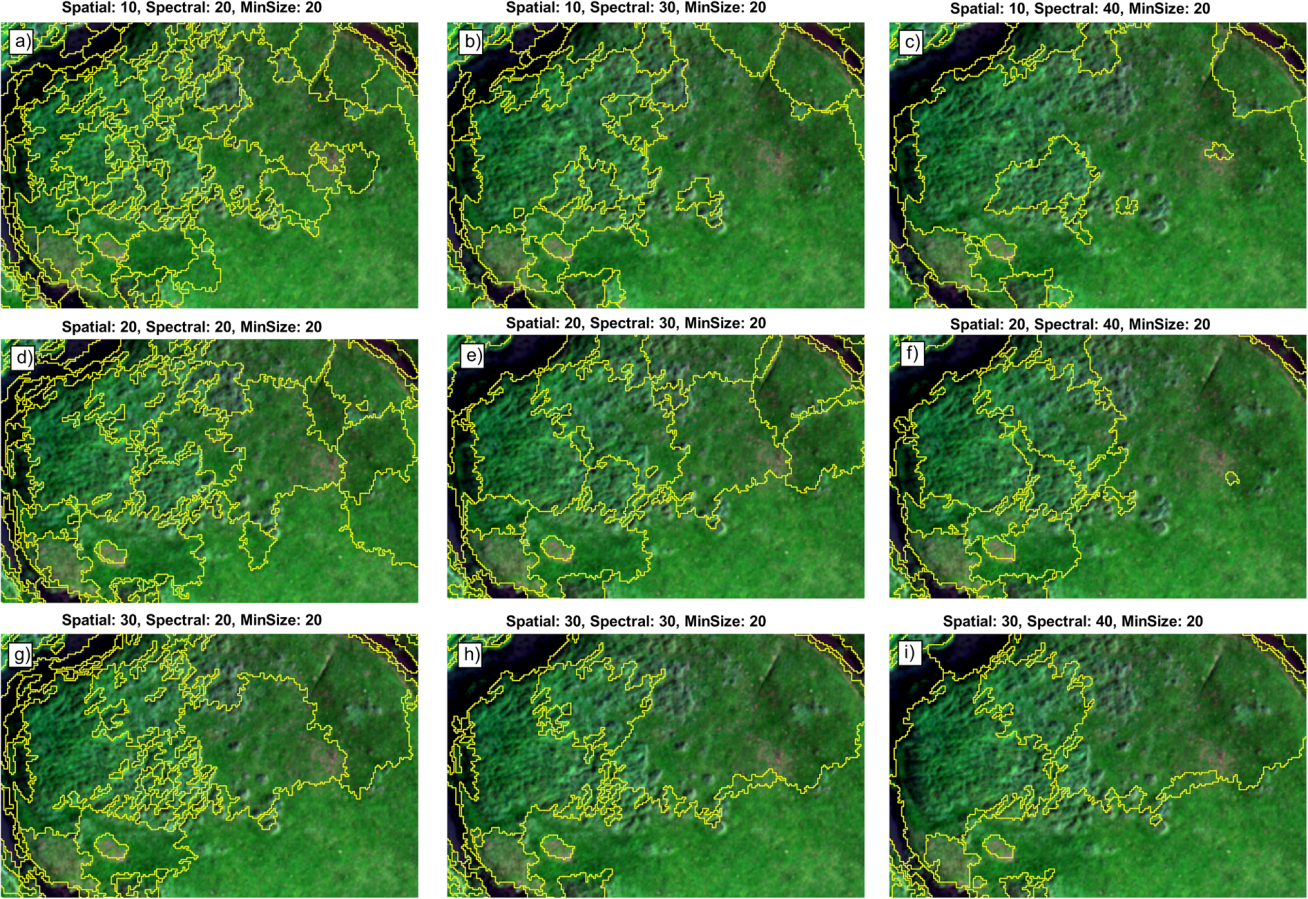
Object-based image segmentation based on changes in the spatial and spectral parameters. The image subset shows the *Elymus* patches (clearly visible in subfigure **i**, water (dark) and saltmarsh (green lawn-like structure)). Yellow lines mark the boundaries of the polygon segments. The best configuration was found for the setting in subfigure **d**

The final step in mapping *Elymus* patches required the classification of the polygon segments into a set of thematic classes which can be found in the study area. The following eight classes were identified: *Elymus athericus*, mixed saltmarsh (mainly *Festuca rubra* and *Puccinellia maritima*), *Limonium vulgare* patches, barren ground, dyke, path, water, and mudflat. For each class, approximately 60–80 in situ locations were identified in the image and digitized as point vectors, except for classes with small spatial extent and homogenous spectral properties, e.g., mudflat or dyke. In these cases, we took between 20 and 30 in situ points. Then, for each of the nine different segmentation model outputs (Fig. 2), polygons were extracted based on the in situ points and were split into training and test datasets with a 70/30 ratio stratified by class.

Three different classification algorithms were tested. First, the random forest algorithm (Breiman 2001) was selected because it is easy to implement, one of the most widely used image classifiers and also one of the most successful algorithms in a recent benchmark study on image classification algorithms (Pirotti et al. 2016). We optimized the algorithm for the *mtry* parameter and used repeated 10-fold cross-validation to identify the optimal model settings. Second, we used the elastic net algorithm (Zou and Hastie 2017). Elastic net provides automatic variable selection via shrinkage technique identifying parameters with low explanatory power, setting their coefficients to nearly zero. Further, elastic net applies regularization to prevent the model from overfitting. Despite its interesting features, elastic net was rarely tested on image datasets. We trained the elastic net with a 10-fold cross-validation but without hyperparameter tuning as this was done automatically by the algorithm. The third classifier we used was the artificial neural network (ANN). ANNs can be considered complex non-linear versions of logistic regression (Venables and Ripley 2002) using a network of hidden layers to learn from the data via feedback loops. Neural networks, in particular deep learning methods, have gained strongly in popularity for image classification, recently (Li et al. 2018). Here, we used the standard feed-forward neural network as implemented in the *nnet* library (Venables and Ripley 2002). We calibrated the network by varying the number of hidden layers from one to ten.

Each classifier was evaluated on an independent test dataset. We measured the performance using three accuracy measures. First is overall accuracy (OA), which measures the general agreement of the predictions with the true class labels. OA is a standard parameter in classification exercises. Second, we use the kappa coefficient (Cohen 1960), which provides information on the agreement of observed versus an expected accuracy. Kappa can be applied to multiclass classifiers to evaluate the overall performance of a classifier. Values of kappa above 0.8 are considered to be excellent (Landis and Koch 1977). Although it is strongly criticized for being correlated with OA and for its philosophy of chance-based agreement (Olofsson et al. 2014), we still use it here as a measure for reference. Finally, we used the F1-single class measure for the class “*Elymus*.” F1 is the harmonic average of two other accuracy measures called precision and recall. F1 is often used when no focus on either precision or recall is required but the classifier should be balanced. Variable importance was measured in percentage of occurrence within the cross-validated models, or in other words, how often a parameter was used relative to the number of all models. All steps during the classification and evaluation were done in the software R v.3.4 (R Core Team, 2019) using the package caret (Kuhn and Johnson 2013).

### Map comparison

In order to evaluate the differences between the field-based polygons and the OBIA-based mapping, a comparison with regard to location, shape, and area in hectare of *Elymus* polygons was done. In addition, we conducted an overlap analysis in QGIS. Polygonal overlap was measured in hectare and percentage. We post-processed the OBIA polygons by removing all polygons which could not be identified as a correct “*Elymus*” patch. For this, we created a hectare grid across the CIR image and checked each polygon whether it contained *E*. *athericus* patches or not. Typical misclassifications involved areas with shadows, water runnels, or small tracks that had a similar texture like the “*Elymus*” class. The overlap analysis was then repeated for the post-processed polygons.

## Results

### Image segmentation

The image segmentation algorithm was tested with nine different parameter settings. Spatial radius and spectral distance ranged between values of 10 and 40, while the minimum size parameter was kept constant at a value of 20 pixels. The outcomes of the different settings are shown for an example location in Fig. 2. An increase of the spatial radius parameter led to larger polygons (Fig. 2a, d, h, g). However, many small polygons remain in the area of the “*Elymus*” patch, which reflect the strongly heterogeneous texture of *Elymus athericus* patches. An increase in the spectral range parameter led to increasingly larger polygons reducing the overall number of small polygons. This resulted in wrongly placed boundaries, in particular for all spectral values of 40 (Fig. 2c, f, i). Low spatial and spectral values (Fig. 2a) yielded many small polygons, being not able to capture the “*Elymus*” patches correctly, while high spatial and spectral values (Fig. 2i) caused too large polygons with wrong boundaries. The average solution (spatial = 20, spectral = 20; Fig. 2d) was later identified as the optimal configuration.

### Object classification

The classification of the polygon segments was done for nine different parameter settings and three different machine learning algorithms: random forest, elastic net, and artificial neural networks. Based on three common accuracy measures, i.e., OA, kappa, F1, we evaluated the performance of the classification outcomes (Table 1). All accuracy measures decreased with increased spectral range parameter values as this led to larger polygon segments (Fig. 2). The increase of spatial radius had a less strong effect. High accuracy was always detected for spatial (sp) = 20, except for the first model (Table 1). Models with sp = 30 or spectral range (sr) = 40 always performed worse. The overall highest accuracy was found for artificial neural network (ANN) with the setting sp = 20, sr = 20 (Table 1), while the worst model had the setting sp = 10, sr = 40. Interestingly, the model that best classified the “*Elymus*” class was the model sp = 10, sp = 20, with F1 = 0.954, followed by the best ANN model with F1 = 0.931. With regard to the algorithms used, there was no algorithm which clearly performed better than the others. However, the ANN model often had the highest overall performance or was at least as good as the others while elastic net always scored last. The elastic net model with sp = 10, sr = 40 was the worst model with very low accuracy values (Table 1). We decided to choose ANN over the random forest as the final model as the other classes were better modeled with the ANN (see F1 in Table 2). Further, the vegetation map produced by ANN appeared more precise when compared to the vegetation map based on the random forest model with sp = 10, sr = 20.

**Table 1:** Overview for the different accuracy measures at different segmentation parameterizations. *OA* overall accuracy, *K* kappa, *F1* F1 measure for the class *Elymus*; # Samples depict the number of training and test polygons. Numbers shown in bold mark the highest value per column. The model with spatial = 20, spectral = 20 was chosen as the optimal model

# Samples		Segmentation parameters		Random forest			Elastic net			Artificial neural network		
Train	Test	Spatial	Spectral	OA	K	F1	OA	K	F1	OA	K	F1
511	230	10	20	**0.943**	**0.928**	**0.954**	0.909	0.884	**0.929**	0.922	0.901	0.886
440	205	10	30	0.902	0.879	0.894	0.863	0.830	0.881	0.878	0.849	0.872
383	186	10	40	0.849	0.814	0.821	0.613	0.509	0.617	0.839	0.801	0.788
543	240	20	20	0.942	0.927	0.917	**0.942**	**0.927**	0.916	**0.950**	**0.937**	**0.931**
493	228	20	30	0.908	0.884	0.901	0.925	0.907	0.915	0.934	0.918	0.915
455	219	20	40	0.904	0.880	0.885	0.913	0.892	0.889	0.927	0.909	0.896
541	238	30	20	0.924	0.904	0.897	0.912	0.888	0.870	0.933	0.915	0.905
506	232	30	30	0.905	0.881	0.885	0.901	0.875	0.877	0.909	0.886	0.884
465	227	30	40	0.890	0.862	0.868	0.894	0.867	0.864	0.894	0.868	0.871

**Table 2:** Confusion matrix for the best classification model (ANN) for spatial = 20, spectral = 20, and minimum size = 20. Columns represent reference class labels while rows represent predicted class labels. The diagonal shows the correct classified number of samples while the off-diagonal values refer to misclassifications. The last row shows the class specific F1 accuracy measures

	Bare soil	Dyke	*Limonium*	*Elymus*	Mixed salt marsh	Water	Mudflat	Path
Bare soil	33	0	0	0	1	0	0	1
Dyke	0	23	0	0	0	0	0	0
*Limonium*	0	0	13	0	0	0	0	0
*Elymus*	1	0	1	61	2	0	0	0
Mixed salt marsh	0	0	0	4	67	0	0	0
Water	1	0	0	1	0	21	0	0
Mudflat	0	0	0	0	0	0	4	0
Path	0	0	0	0	0	0	0	6
Precision	0.919	1.000	1.000	0.910	0.956	0.913	1.000	1.000
Recall	0.971	1.000	0.929	0.924	0.929	1.000	0.750	0.857
F1	0.943	1.000	0.963	0.931	0.950	0.954	1.00	0,929

The confusion matrix for the ANN model (Table 2) showed that *Elymus* samples were partly confused with the class “mixed saltmarsh,” “bare soil,” or “water.” Classes that were not “*Elymus*” but were classified as “*Elymus*” were “mixed saltmarsh,” “bare soil,” and “*Limonium*.” No other large misclassifications were observed indicating a good performance of the ANN model. Several classes had a low number of test samples, e.g., for “*Limonium*,” “mudflat,” and “path,” but were still well classified with F1 values over 0.9. The class “path” was the worst performing class but still had an accuracy of 0.929.

The importance of the image parameters did not differ much between the different algorithms. The vegetation indices were always more important than the textural parameters and the canopy height was in between. The mean values and the standard deviations of NDVI, NDWI, and NIR had always importance values above 75%. The textural values were always below 40%. Due to the different numbers of cross-validation runs, the importance of the parameters cannot be directly compared. Also, the output of the elastic net algorithm did not provide an overall importance but only class-wise importance values. Hence, we only give this rather coarse interpretation of parameter importance. In sum, the ratio of spectral versus textural importance values is approximately 75:25 for all algorithms used.

### Comparison of field survey– and OBIA-based mapping of *Elymus athericus*

We compared the vegetation mapping of *Elymus athericus* dominated stands versus the OBIA-based map of *E*. *athericus* polygons for visual and spatial agreement. Considering the classification, both mapping techniques resulted in similar area sizes. According to the field survey, 6.29 ha were covered by the vegetation type *Elymus athericus*, while the OBIA-based approach resulted in 6.22 ha, or 35.0% and 34.6% of the study area. However, the overlap between both polygon datasets was only 70% or 4.2 ha. When the mapped polygons were weighted by the cover estimates per polygon, the weighted *E*. *athericus* area was slightly smaller with 4.77 ha (26.5%) of the study area. This roughly corresponds to the overlap of OBIA and field survey polygons with 4.2 ha.

**Fig. 3:**
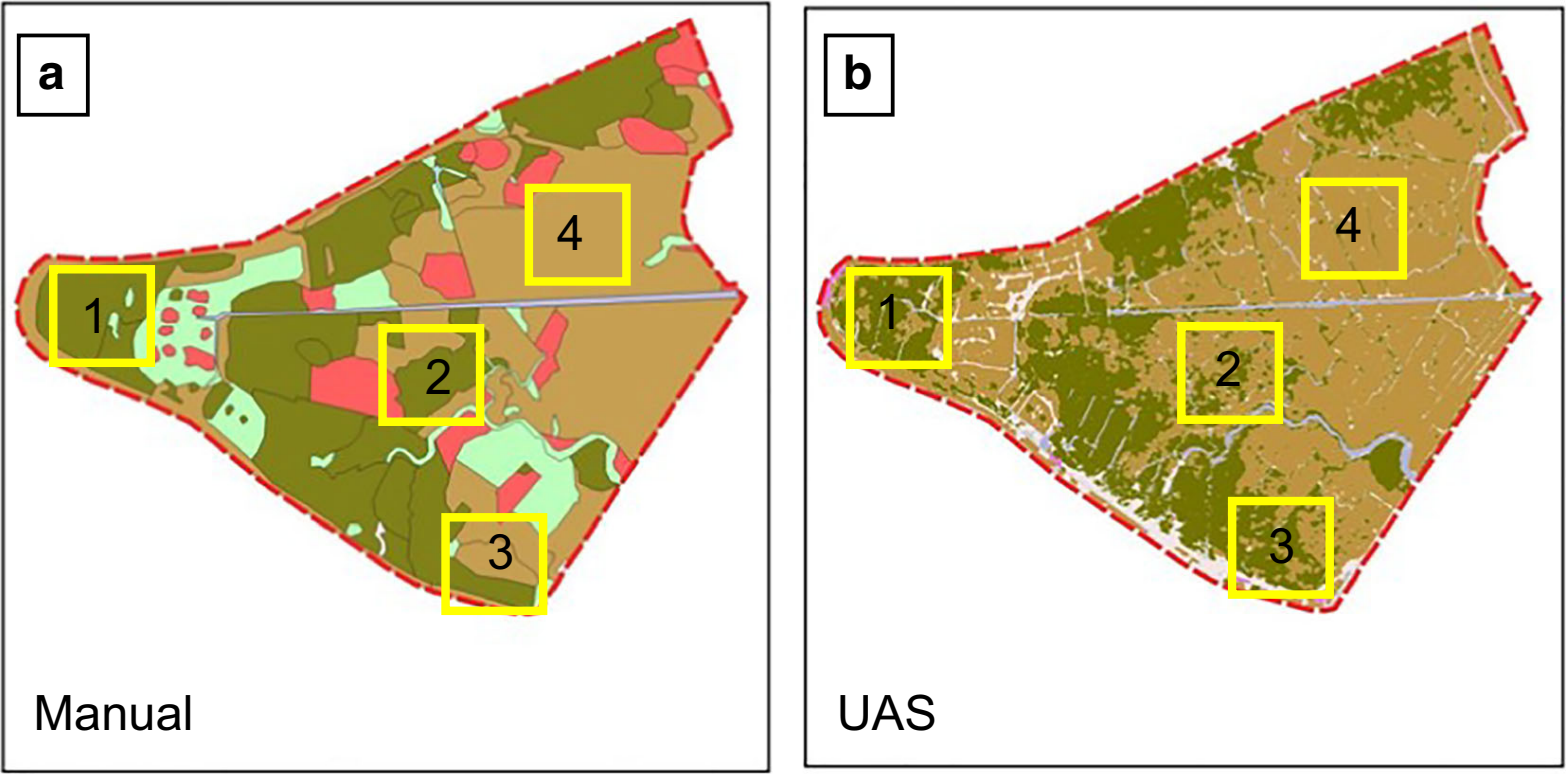
Comparison of manual (**a**) and OBIA-based (**b**) mapping results. The *Elymus* patches are colored in olive green, while saltmarsh is colored light-brown. Other vegetation units in **a** are dominated by *Festuca rubra* (red) or *Puccinellia maritima* (light green). Water runnels are light-blue. The yellow boxes highlight areas of difference between the two approaches

A visual comparison (Fig. 3) revealed that manual mapping commonly overestimated the extent of *E. athericus* patches locally. For example, in Fig. 3a, Boxes 1 and 2 contain large polygons outlined by the manual approach, yet the OBIA-based map (Fig. 3b) show many fine-structured patches in the same area. Box 3 shows a sharp boundary drawn with the manual approach; however, the OBIA-based map extends in this area more accurately. Here, the OBIA-based approach found many small singular patches, while the manual approach, which required a coverage of at least 30% to be classified as the *E*. *athericus* vegetation type, ignored this part. On the other hand, the OBIA-based map also produced many false-positive patches by identifying small water runnels or shadows as *E*. *athericus* patches in the large area covered by mixed saltmarsh which is generally free of *E*. *athericus* (box 4). Hence, the general pattern of the distribution of *E*. *athericus* is in agreement between the two methods; however, on a finer spatial scale, OBIA led to finer and more accurate delimitation of *E*. *athericus* patches but also to many small false areas.

After post-processing the OBIA-based polygons, many were eliminated in the eastern part of the study area and on the dyke, leaving 5.91 ha of *E. athericus* cover. This was equal to a difference of 0.31 ha in comparison to the original OBIA-based polygons and 0.38 ha compared to the field-based polygons. The overlap of the post-processed polygons and the field-based polygons did only change marginally by 1%; hence, most of the area that was wrongly classified as *E*. *athericus* was in areas near boxes 3 and 4 (Fig. 3).

## Discussion

Drones, or UAS (unmanned aerial systems), have seen frequent successful applications in conservation biology for mapping endangered or problematic plant species in wetland ecosystems over the last years. In this study, we successfully conducted a UAS flight campaign in a saltmarsh ecosystem, classified the area covered by the tall grass *Elymus athericus* and compared our results to the distribution of *E. athericus* mapped by a field survey in the study area.

We found that field-based maps of *E*. *athericus* patches were in good spatial agreement with the OBIA-based maps, showing the same amount of area covered by *Elymus*. However, we found that field-based maps were rather based on few large polygons that also covered areas where *E*. *athericus* was not present, thus overestimating its total cover. The OBIA-based maps instead revealed a more realistic picture consisting of many single polygon fragments, thus better delineating local patches. However, OBIA-based maps also overestimated *E*. *athericus* in areas where the species was not observed by the field-based survey and where it was not present. The area difference between the two maps was 30% or ca. 2 ha which is a significant amount. The study done by Tay et al. (2018) also found good agreement between field-based cover estimates and cover estimates of a pixel-based classification (*R*^2^ = 0.79) of northern German meadow vegetation, specifically targeting *Jacobaea vulgaris*. However, they found that cover of *J*. *vulgaris* was systematically underestimated which was mainly caused by different ways of measuring cover (the rosette was included in the field survey, but the UAS only measured the flower head). In a different context, Hill et al. (2017) compared three methods, i.e., field survey, manual digitization, and pixel-based classification of UAS-images, to map *Iris pseudacorus* at two lakes in British Columbia, Canada. They found very little overlap of only 7% between the field survey and the classified drone-based maps. This was caused by the difficulties of correctly locating the Iris populations in the GIS as the ground truth was gathered from canoe. The manual digitization was considered the most accurate but also time-consuming method. These studies suggest that drone-based imagery does not yield optimal results when compared to ground cover estimates, as such comparisons are difficult to achieve. Our findings that the OBIA-based map mainly overestimates in areas where *E*. *athericus* did not occur, but yields a more realistic picture in areas where field-based polygons overestimate, are promising. The error caused by many small false-positive polygons can be easily cleaned by either fine-tuning the OBIA method, deleting polygons based on unique image features, or manually deleting superfluous polygons. Although this will add to the required overall processing time, it will also increase the credibility and usability of the final map. In sum, image segmentation using the large-scale mean shift segmentation algorithm (LSMS, Michel et al. 2015) with varying levels of spatial and spectral thresholds on a VHR-multispectral drone imagery was of higher accuracy in terms of delineation of *E*. *athericus* patches when compared to field-based maps.

The quality of the classification of the image segments resulting from the object-based image analysis (OBIA) ranged for kappa between 0.51 and 0.94. We compared three different types of classifiers, i.e., artificial neural networks, elastic net, and random forest. We found no other study that compared the elastic net as a classifier for remotely sensed imagery, but it was constantly the worst in our study. It is unclear why its performance was much lower than the others. One possible explanation is that it allowed only the tuning of the regularization parameter lambda, which might have not been flexible enough. The other two classifiers (ANN, RF) were on par. Many other studies found that ANN and even more often RF were very good classifiers (Poona and Ismail 2013; Chabot et al. 2016; Oldeland et al. 2017, Silveira et al. 2019). Hence, these can be recommended for future studies. However, few studies presented a thorough examination of the parameterization of the LSMS spatial-spectral thresholds as we did (e.g., Gonçalves et al. 2019). Often studies report fixed threshold values according to object size and its spectral variation. For example, Sosa-Herrera et al. (2019) used the same LSMS algorithm as we did, and mapped the health status of *Capsicum annuum* crops using a UAS. They set a fixed spatial threshold of 5 and the spectral threshold to 15 based on estimated values. Another study on vegetation of cork oak woodland (De Luca et al. 2019) found that values for the spatial threshold above 10 made the calculations very time consuming and thus skipped the mean shift smoothing-step in the image segmentation workflow. We run our LSMS on a standard PC with eight cores and 8 GB RAM; the script containing command lines for nine models was written in a batch file and all calculations ran within 12 h. The classification step took about 30 min for the 27 different models. In contrast to manually comparing parameter values, the batch script containing different parameter settings greatly optimized the LSMS workflow. We recommend using such a grid-wise tuning approach to optimize thresholds for future studies that apply image segmentation on the UAS imagery.

The methodology described in this study allowed us to map *Elymus athericus* with high accuracy with two 30-min UAS flights. Although other studies argued that UAS-based campaigns are quick but require a lot of time for image processing and classification (Hill et al. 2017; Tay et al. 2018), we found that these steps can be done with a reasonable effort. Of course, the general workflow has to be established in terms of batch files containing command line code and R-scripts for running the classification. But once established, the workflow can then be applied on any scene; hence, especially for ongoing monitoring projects, it is worth the investment. A similar approach was invented with the SegOptim-R package (Gonçalves et al. 2019), which provides such a workflow as a ready-to-use open-source software package. Once established, the classification algorithm based on the training data can be applied to image data collected by the same camera sensor at new sites or new time points, and the resulting maps can improve the information base for environmental management. Thus, our approach provides support for long-term, repetitive monitoring while optimizing costs and field surveys. This is particularly important for sites of low accessibility such as wetlands (Boon et al. 2016) or when vegetation becomes relatively small in comparison to the landscape scale (Turner et al. 2019). Minimizing in situ mapping and control operations is a great advantage of using UAS-based remote sensing strategies. In general, this approach can allow environmental managers to monitor the distribution and abundance of plants in similar situations once the classification models are adjusted to the respective species. Moreover, the development of new, lightweight multispectral sensors using on-board radiometric calibration encourages the transferability of classification models to other areas.

However, some limitations of our approach also became obvious. Besides the many technical challenges, such as calibration, image registration, and processing, which are discussed in detail elsewhere (Manfreda et al. 2018), we will focus on three more applied challenges. First, there is a clear discrepancy between field-based and OBIA-based polygons in terms of spatial resolution and also in area statistics. Thus, there is no direct way of converting the obtained values from one method to the other and care needs to be taken if ongoing field-based monitoring is to be replaced by pure UAS-based monitoring. Second, post-processing of image segmentation is usually required due to obvious misclassifications by the algorithm, in this case in the area of low growing mixed saltmarsh. This may be the case even though the accuracy evaluation shows very high rates of agreement between train and test data. Consequently, a fully automatic approach is hardly achievable and expert judgment will always play a role in post-processing of image segments. Third, there is clearly a lack of standardization for UAS-based vegetation mapping efforts. There are many studies that describe the necessary steps to set up a UAS mission (e.g., Tmušić et al. 2020), but actually very little agreement was reached on how to appropriately analyze the very-high-resolution imagery made available by these UAS missions. Clearly this is complicated by the many different possible applications. Here, we proposed an object-based image segmentation approach, from which we believe it is highly suited for the task of mapping large conspicuous patches of a single plant species. The recent advent of deep learning methods for VHR-image classification might further improve and facilitate mapping of vegetation (Kattenborn et al. 2019) potentially making OBIA methods superfluous.

## Conclusion

Drones or UAS offer capabilities for mapping the occurrence, dynamic or extent of plant species in a given conservation management setting with high accuracies. UAS-acquired very-high resolution image data generate additional valuable geospatial information for management just in time when needed at relatively low cost. We were able to map the distribution of the grass species *Elymus athericus* in a managed saltmarsh and compared field- and UAS-based maps of the species distribution. At the coarse scale, the patterns of both approaches were in good agreement. However, on a finer scale, the field-based maps overestimated coverage where *E*. *athericus* was present, while the UAS-based maps overestimated coverage in areas where the species was generally absent. After post-processing, the UAS-based maps yielded an area estimate of about 1–2 ha less compared to the coarse polygons resulting from manual, field-based mapping. Furthermore, we found that the classification of vegetation using object-based image segmentation can lead to very high accuracies, but a comparison of different classification algorithms and optimally parameterized thresholds for the segmentation algorithm is of high importance. Once the workflow is established, UAS-based surveys significantly increase the objectivity of long-term monitoring of plant species distributions with comparable effort. The main advantages of the drone-based workflow are the objectivity, reproducibility in the next monitoring interval, just-in-time produced images from the same point in time as the ground controls or field surveys are carried out, and images of the study area with a much higher resolution than satellite or conventional aerial imagery can provide. As such, we are convinced that UAS-based remote sensing techniques will be integrated step by step in applied conservation management.
